# Processes for updating guidelines: protocol for a systematic review

**DOI:** 10.12688/hrbopenres.13448.1

**Published:** 2021-11-01

**Authors:** Karen Cardwell, Joan Quigley, Barbara Clyne, Barrie Tyner, Marie Carrigan, Susan Smith, Máirín Ryan, Michelle O'Neill

**Affiliations:** 1 Health Technology Assessment Directorate, Health Information and Quality Authority, Dublin, Ireland; 2 Department of General Practice, Royal College of Surgeons in Ireland, Dublin, Ireland; 3 Department of Pharmacology & Therapeutics, Trinity College Dublin, Dublin, Ireland

**Keywords:** Systematic review, guideline methodology, guideline update, prioritization methodology.

## Abstract

**Background:** National Clinical Guidelines are systematically developed statements, based on a thorough evaluation of the evidence, to assist practitioner and service users’ decisions. Clinical guidelines require updating to ensure validly of the recommendations contained within. The purpose of this systematic review is to describe the most recent guideline update processes, including prioritisation methods, used by international or national groups who provide methods guidance for developing and updating clinical guidelines.

**Methods:** A combination of searching a pre-defined list of international and national organisations that provide methods guidance for developing and updating clinical guidelines, together with grey literature searching, will be undertaken to identify relevant handbooks. This will be supplemented by a systematic literature search of Medline (EBSCO), Embase (OVID) and The Cochrane Methodology Register. As guideline development methodology has evolved considerably, the overall search span for this systematic review will be the last 10-years (2011–2021). Publications eligible for inclusion are methodological handbooks that provide updating guidance, including prioritisation methods, for clinical practice guidelines and peer-reviewed articles that describe or have implemented updating guidance, including prioritisation methods. Using Covidence, two reviewers will independently review titles/abstracts and full texts. Where disagreements occur, discussions will be held to reach consensus and where necessary, a third reviewer will be involved. Methodological handbooks will be quality assessed (using the GIN-McMaster Guideline Development Checklist) independently by two reviewers and any disagreements will be resolved by deliberation, or if necessary, a third reviewer. Data will be extracted by one reviewer and checked for inaccuracies/omissions by a second. A narrative synthesis will be undertaken.

**Conclusions:** Updating clinical guidelines is an iterative process that is both resource intensive and time-consuming. The findings of this systematic review will support clinical guideline developers to ensure appropriate investment of resources.

## Abbreviations

CG, Clinical guideline; CIMO, Context, intervention, mechanism, outcome; GIN, Guidelines International Network; NCG, National Clinical Guideline; PRISMA-P, Preferred Reporting Items for Systematic Reviews and Meta-Analyses Protocols.

## Introduction

In Ireland, the National Clinical Effectiveness Committee (NCEC), established in September 2010, works to prioritise and quality assure National Clinical Guidelines (NCGs) so as to recommend them to the Minister for Health to become part of a suite of NCGs ^1^. Clinical guidelines (CGs) are systematically developed statements, based on a thorough evaluation of the evidence, to assist practitioner and service users’ decisions about appropriate healthcare for specific clinical circumstances across the entire clinical system ^1^. The recommendations contained within CGs are underpinned by evidence syntheses, that is, systematic reviews or adaptation of existing CGs and or recommendations ^2^. Ongoing evolution of the scientific literature brings the emergence of new evidence which can change the findings of a systematic review and, as a consequence, change the recommendations made within a CG. As such, CGs need to be updated regularly to ensure validity of the recommendations contained within ^3^.

Updating CGs is an iterative process that is both resource intensive and time-consuming. Typically, CGs are updated in accordance with a pre-defined time-period. For example, the National Institute for Health and Care Excellence ^4^, American College of Physicians ^5^ and US Preventive Services Task Force ^6^ indicate that CGs should be updated every five years; in Ireland, the National Clinical Effectiveness Committee advises updating CGs every three years ^1^. However, it is also acknowledged that deciding to update a CG depends on factors other than pre-defined time periods, such as the volume and potential impact of new research published on the topic, clinical burden of the topic, economic impact and resources available to update a guideline ^1^. For that reason, policy makers and other stakeholders are advocating for a move away from updating guidelines based on a pre-defined time-period and moving towards updating guidelines based on prioritisation criteria, to ensure appropriate investment of resources ^7^.

Just as evolution of the scientific literature brings new clinical evidence that can impact the recommendations within a CG, it also brings advancement in methodologies used in development and updating of CGs ^3^. One such advancement has been the emergence of rapid and living guidelines which aim to provide timely advice for decision-makers by optimising the guideline development process whereby individual recommendations can be updated as soon as new relevant evidence becomes available ^8^. The use of rapid and living guidelines has been especially evident throughout the COVID-19 pandemic where the emphasis was on development and implementation of strategies to manage the rapidly evolving evidence base in response to a public health emergency ^9^.

Previous systematic reviews on this topic have summarised guidance from methodological handbooks for updating clinical practice guidelines ^3^, strategies for prioritisation of clinical guidelines that require updating ^7^ and prioritisation processes for the de novo development, updating or adaptation of guidelines ^10^. However, the evidence synthesised was largely published over a decade ago and related to update processes developed for a particular disease-specific guideline or specifically to updating systematic reviews, not updating clinical guidelines.

Therefore, the purpose of this systematic review is to describe the most recent guideline update processes, including up-to-date prioritisation methods, used by international or national groups who provide methods guidance for developing and updating clinical guidelines. The focus of this systematic review was not on adaptation, contextualization, or de novo development of guidelines, but instead updating processes for existing guidelines. This will support guideline development groups nationally and internationally in consideration of amendments to the current update processes.

## Methods

Details of this protocol have been submitted to the PROSPERO database (registration number: CRD42021274400). Any amendments made to the protocol will be acknowledged on PROSPERO and in any subsequent publications. This protocol outlines the proposed approach to achieve the stated purpose and has been informed by the Preferred Reporting Items for Systematic Review and Meta-Analysis Protocols (PRISMA-P) guidelines ^11^.

### Criteria for considering publications for this review

This systematic review protocol has been developed to answer the review question:

*What are the most recent guideline update processes, including up-to-date prioritisation methods, used by international or national groups who provide methods guidance for developing and updating clinical guidelines?*

The review question was formulated in line with the CIMO (Context, Intervention, Mechanism, Outcome) framework ^12^, as presented in Table 1. The CIMO framework describes “the problematic Context, for which the design proposition suggests a certain Intervention type, to produce, through specified generative Mechanisms, the intended Outcome(s)” ^12^. The context describes the environment within which change occurs, the intervention is what influences a change, the mechanism is triggered by the intervention and this produces the outcome ^12^.

**Table 1.** Context, Intervention, Mechanism, Outcome.

**Context**	▪ Clinical guidelines require updating to maintain relevancy.
**Intervention**	▪ International or national groups provide methods guidance (in published handbooks and/or peer-reviewed articles) for developing and updating clinical guidelines, as well as prioritising clinical guidelines for updating.
**Mechanism**	▪ Clinical guidelines considered for updating (includes full, modular, rapid updates). ▪ Tools or guidance available to support prioritisation.
**Outcome**	▪ Description of update (or retirement) process (including roles and responsibilities at each stage) ○ types of update that exist ○ criteria used to determine if update necessary ○ process for retiring a guideline ○ criteria to prioritise which guideline is updated first ○ criteria to prioritise which clinical questions within a guideline are updated ○ evidence synthesis methodologies used to update clinical questions ○ dissemination of updated guideline ○ resources required to undertake update ○ differences between review process for updated guideline verses original guideline ○ differences between approval and endorsement process for updated guideline versus original guideline ▪ Evaluation of the process ○ usability and or critique of the updating methodology ○ timeliness, that is, specific processes that enable a more efficient and timely update.

The types of publications eligible for inclusion will be:

▪ methodological handbooks that provide updating guidance, including prioritisation methods, for clinical practice guidelines
▪ peer-reviewed articles that describe or have implemented updating guidance, including prioritisation methods.

Only publications from 2011 onwards will be considered for inclusion; publications published before 2011 will have been included in the index documents ^3, 7^ but will not be included in this review.

***Exclusion criteria.*** The following exclusion criteria will be applied:

▪ Due to issues relating to transferability of guidelines developed for specific diseases, disease-specific publications (handbooks and or peer-reviewed publications which describe, or have implemented, guidance for updating disease-specific guidelines).
▪ Editorials/commentaries/opinion pieces.
▪ Abstracts only.
▪ Animal studies.
▪ Non-English language publications.

### Search methods for identification of studies

Due to changes in process and methodologies in guideline development in the last 10 years, the overall search span for this review will be the last 10-years (2011–2021). The primary data source for this review will be methodological handbooks which detail update processes, including prioritisation methods, used by international or national groups who provide methods guidance for developing and updating clinical guidelines. Through scoping searches, we identified a published systematic review of methodological handbooks that provide guidance for updating clinical practice guidelines ^3^. This systematic review by Vernooij *et al.* ^3^ was published in 2014 and will be considered an index document, whereby for methodological handbooks, data from 2011–2012 will be taken from Vernooij *et al.* ^3^ and data from 2013–2021 will be gathered through a new search of organisations’ websites and grey literature.

The secondary data source will be peer-reviewed articles which detail the development of, and or evaluation of guideline update processes. For peer-reviewed articles, data from 2011–2021 will be gathered through a database search. While peer-reviewed articles will not be the primary data source for this systematic review, they may serve as “sign-posts” to the handbooks and may also provide qualitative data relating to the usability of the handbooks and update processes.

In 2017, Martinez-Garcia *et al.* ^7^ published a systematic review of prioritisation processes for updating guidelines which focused on peer-reviewed articles rather than methodological handbooks. Data specific to prioritisation methods from 2011–2015 will be taken from Martinez-Garcia *et al.*, ^7^ and data from 2016–2021 will be gathered from the new database search.

***Organisations.*** The websites of organisations listed in Table 2 will be searched for relevant methodological handbooks. The organisations were chosen based on advice from the Clinical Effectiveness Unit of the Department of Health (which supports the work of the National Clinical Effectiveness Committee), and or identification of the organisation from previous systematic reviews on this topic and guidance being available in English.

When guideline manuals are not found online, or where any data gaps are identified, these will be addressed by contacting organisations (via email) to gather information relating to guideline update processes (including prioritisation methods). Other relevant organisations identified during the searching process will also have their website searched.

**Table 2.** Organisations that will be searched for relevant methodological handbooks.

Organisation name	Organisation URL
Agency for Healthcare Research and Quality, USA	https://www.ahrq.gov/
Australian National Health and Medical Research Council, Australia	https://www.nhmrc.gov.au/
Belgian Health Care Knowledge Centre, Belgium	https://kce.fgov.be/en
Canadian Agency for Drugs and Technologies in Health, Canada	https://www.cadth.ca/
European Network for Health Technology Assessment	https://www.eunethta.eu/
Finnish Institute for Health and Welfare, Finland	https://thl.fi/fi/
Guidelines International Network	https://g-i-n.net/
Institute of Medicine, USA	https://nam.edu/about-the-nam/
McMaster GRADE centre, Canada	https://cebgrade.mcmaster.ca/
National Institute for Health and Care Excellence, UK	https://www.nice.org.uk/
Ravijuhend, Estonia	https://www.ravijuhend.ee/
Scottish Intercollegiate Guidelines Network, Scotland	https://www.sign.ac.uk/
National Board of Health and Welfare, Sweden	https://www.socialstyrelsen.se/en/regulations- and-guidelines/national-guidelines/
Public Health Agency of Sweden, Sweden	https://www.folkhalsomyndigheten.se/the- public-health-agency-of-sweden/
The Best Practice Advocacy Centre New Zealand, New Zealand	https://bpac.org.nz/guidelines/
World Health Organization	https://www.who.int/

***Grey literature.*** Other sources of grey literature will be searched for relevant methodological handbooks. These are listed in Table 3.

**Table 3.** Grey literature that will be searched for relevant methodological handbooks.

Grey literature source	URL
Google (first 10 pages of results)	www.google.com
Open Grey	http://www.opengrey.eu/
Reference chasing	NA

***Databases.*** The following databases will be searched for peer-reviewed articles using the search strategy defined in Supplementary file 1:

▪ Medline (EBSCO)
▪ Embase (OVID)
▪ The Cochrane Methodology Register

### Selection of eligible publications

Methodological handbooks will be identified through searching the websites of eligible organisations (see Table 2) and through screening the methodological handbooks included in the index document ^3^. This will be done by one reviewer and relevant handbooks will be imported into Endnote (Version X8). Imported handbooks will be reviewed by a second reviewer to confirm their eligibility.

All citations identified from the collective search strategy (see Supplementary file 1), and through screening the peer-reviewed articles included in the index document ^7^, will be exported to EndNote (Version X8) for reference management, where duplicates will be identified and removed. Using Covidence ( www.covidence.org), two reviewers will independently review the titles and abstracts of the remaining citations to identify those for full-text review. The full texts will be obtained and independently evaluated by two reviewers applying the defined inclusion and exclusion criteria. Where disagreements occur, discussions will be held to reach consensus and where necessary, a third reviewer will be involved. Citations excluded during the full-text review stage will be documented alongside the reasoning for their exclusion and included in the PRISMA flow diagram.

### Data extraction and management

Data will be extracted from methodological handbooks by one reviewer and checked for accuracy and omissions by a second. Where disagreements occur, discussions will be held to reach consensus and where necessary, a third reviewer will be involved. Data extraction will be conducted in Microsoft Word, using a data extraction form (Supplementary file 2). The data extraction form will be piloted first and refined as necessary.

Relevant data to be extracted will include:

▪ the types of update that exist, for example partial or full
▪ criteria used to determine if an update is necessary
▪ process for retiring a guideline
▪ criteria used to prioritise which guideline to update first
▪ criteria used to prioritise clinical questions to be updated within a guideline
▪ evidence synthesis methodologies used to update clinical guideline and clinical questions
▪ dissemination of updated clinical guideline
▪ resources required to undertake update
▪ process of reviewing the updated guideline
▪ process of approving and endorsing the updated guideline.

Peer-reviewed articles will not be the primary data source for this systematic review; the primary data source is most likely to be the methodological handbooks. However, in addition to signposting to methodological handbooks, and providing supplemental data relating to update and prioritisation processes, peer-reviewed articles may also provide usability and process evaluation data (relating to the associated handbook); these data will be extracted (see Supplementary file 2).

### Quality assessment

Methodological handbooks will be quality assessed independently by two reviewers and any disagreements will be resolved by deliberation, or if necessary, a third reviewer. In the absence of an appropriate quality assessment tool that is specific for methodological handbooks or guidance, quality will be assessed using the GIN-McMaster Guideline Development Checklist, which is a checklist of items to consider during the development of guidelines. Specifically, we will use the six criteria relating to updating guidelines ^13^. Reviewers will assess, that the methodological handbook covers the following areas:

**1.**  Addresses policy, procedure and timeline for routinely monitoring and reviewing whether the guideline needs to be updated.
**2.**  Addresses who will be responsible for routinely monitoring the literature and assessing whether new significant evidence is available.
**3.**  Sets the conditions that will determine when a partial or a full update of the guideline is required.
**4.**  Makes arrangements for guideline group membership and participation after completion of the guideline.
**5.**  Addresses plans for the funding and logistics for updating the guideline in the future.
**6.**  Addresses documentation of the plan and proposed methods for updating the guideline to ensure they are followed ^13^.

Methodological quality of peer-reviewed articles will be independently assessed by two reviewers. Depending on study design an appropriate version of the Newcastle-Ottawa Scale ^14^ or the Appraisal tool for Cross-Sectional studies (AXIS) ^15^ will be used. The tools will be piloted first on a small number of included studies, and modifications made if needed, before standardising for the remaining studies. Any disagreements will be resolved by deliberation or, if necessary, a third reviewer.

### Data synthesis

As the main data to be extracted for this review is descriptive in nature a narrative synthesis will be undertaken. Data will be summarised under the following headings:

▪ Description of update (or retirement) process (including roles and responsibilities at each stage)
▪ Evaluation of the process.

### Dissemination

The PRISMA checklist will be used to report findings of the review. We will communicate the findings to the NCEC to inform updating processes in Ireland. Findings will also be communicated by publication in a peer-reviewed journal, and by participation in scientific meetings and national and international conferences.

### Study status

Agreement on the study protocol, searching of organisations, grey literature and databases, data extraction and quality assessment is complete. Data synthesis is ongoing.

## Discussion

Updating clinical guidelines is an iterative process that is both resource intensive and time-consuming. This systematic review will summarise the most recent updating and prioritisation processes for clinical guidelines. The findings will support guideline development organisations nationally and internationally to ensure appropriate investment of resources. It will support them in considering and or modifying their current methodologies for updating clinical guidelines. This will be of particular interest in light of new and updated methodologies that have been especially evident throughout the COVID-19 pandemic.

## Data availability

### Underlying data

No data are associated with this article.

### Extended data

Figshare: Supplementary files, https://doi.org/10.6084/m9.figshare.16723063.

This project contains the following extended data:

- Supplementary file 1: Search strategy
- Supplementary file 2: Data extraction tables

### Reporting guidelines

Figshare: PRISMA-P Checklist for “Processes for updating guidelines: protocol for a systematic review”, https://doi.org/10.6084/m9.figshare.16723021.

Data are available under the terms of the Creative Commons Attribution 4.0 International license (CC-BY 4.0).

## Authors’ contribution

KC: Conceptualization, Methodology, Writing – Original Draft Preparation; JQ: Conceptualization, Methodology, Supervision, Writing – Original Draft Preparation; Writing – Review & Editing; BC: Conceptualization, Methodology, Writing – Original Draft Preparation; Writing – Review & Editing; BT: Methodology, Writing – Original Draft Preparation; Writing – Review & Editing; MC: Methodology, Writing – Review & Editing; SS: Writing – Review & Editing; MR: Writing – Review & Editing; MON: Conceptualization, Methodology, Supervision, Writing – Review & Editing.
