# Supplementary material for: Processes for updating guidelines: protocol for a systematic review
Source: HRB Open Res. 2026 Oct 28;4:116. Originally published 2021 Nov 1. [Version 2] doi: 10.12688/hrbopenres.13448.2 (PMC13743837; doi:10.12688/hrbopenres.13448.2)
Supplement: Peer review history

# Reviewer report: Reviewer response for version 2

**DOI:** 10.21956/hrbopenres.16076.r58900

**Published:** 2026-11-06

**Authors:** Julian Little

**Affiliations:** 1 School of Epidemiology and Public Health, University of Ottawa, Ottawa, ON, Canada

I have reviewed the changes and approve. One comment is that it seems weird to be publishing a revised protocol after publishing the systematic review that followed the original protocol. I would welcome comment by the authors and/or the HRB Open Research editorial team on that.

Is the study design appropriate for the research question?

Yes

Is the rationale for, and objectives of, the study clearly described?

Yes

Are sufficient details of the methods provided to allow replication by others?

Yes

Are the datasets clearly presented in a useable and accessible format?

Not applicable

Reviewer Expertise:

NA

I confirm that I have read this submission and believe that I have an appropriate level of expertise to confirm that it is of an acceptable scientific standard.

# Reviewer report: Reviewer response for version 2

**DOI:** 10.21956/hrbopenres.16076.r58946

**Published:** 2026-11-05

**Authors:** Jako Burgers

**Affiliations:** 1 Maastricht University, Maastricht, Limburg, The Netherlands

This protocol is a second version of a protocol for a systematic review on processes for updating clinical guidelines; the original version was published in 2021. The topic is highly relevant for guideline programs with limited resources. Guidelines can become outdated due to a lack of awareness regarding new scientific evidence, professional developments, or changing patient needs. Delays in the updating process can be harmful to patients and society. Consequently, new insights into methods for updating guidelines are welcome. 

 I have the following comments:

 1. The original version of the protocol was published in 2021. It is unclear whether the proposed systematic review has been conducted and if not, why?

 2. An old definition of guidelines is used. In 2011, the IOM changed its definition in ‘Statements that include recommendations intended to optimize patient care that are informed by a systematic review of evidence and an assessment of the benefits and harms of alternative care options’. See https://www.ncbi.nlm.nih.gov/books/NBK209546/ .

 3. The goal of the systematic review is to assess the most recent guideline processes. The search covers guidance and articles from 2011 onwards, which aligns with the initial version of the protocol. This also requires an update, for instance to 2016, to ensure that the data are no more than ten years old.

 4. The use of generative AI (GenAI) is not mentioned, even though it represents a significant development that could resolve certain challenges associated with updating guidelines. Processes could change fundamentally if AI is deployed effectively and efficiently. Specific search terms related to AI could be incorporated into the search strategy.

 5. A clinical guideline provides recommendations or answers to a variety of questions. The need for an update often varies for each individual recommendation within a guideline. To save resources, guidelines can be updated partially. While this aspect is addressed during data extraction and assessment, it may warrant additional attention in the discussion section.

Is the study design appropriate for the research question?

Yes

Is the rationale for, and objectives of, the study clearly described?

Yes

Are sufficient details of the methods provided to allow replication by others?

Yes

Are the datasets clearly presented in a useable and accessible format?

Not applicable

Reviewer Expertise:

Development, implementation, and evaluation of clinical practice guidelines

I confirm that I have read this submission and believe that I have an appropriate level of expertise to confirm that it is of an acceptable scientific standard, however I have significant reservations, as outlined above.

# Reviewer report: Reviewer response for version 1

**DOI:** 10.21956/hrbopenres.14656.r39498

**Published:** 2024-05-21

**Authors:** Brenda Wilson

**Affiliations:** 1 Memorial University of Newfoundland, St. John's, Newfoundland and Labrador, Canada

**Processes for updating guidelines: protocol for a systematic review.**

 Cardwell K, Quigley J, Clyne B, Tyner B, Carrigan M, Smith S, Ryan M, O’Neill M.

 This manuscript describes the protocol for a systematic review of the methods used, by organisations developing clinical guidelines (CGs), for updating their recommendations. This review will be useful because CGs are dependent on an evidence base that may be changing in significant ways, with implications for health outcomes and resource use. Updates themselves require significant resources, so understanding how to prioritise them and carry them out efficiently and to good effect is important.

 The proposed review will focus on the most recent timeframe, and include guidance by major CG organisations internationally as well as peer reviewed reports. The analysis will be descriptive and narrative and offer useful insights to users, funders and those who develop CGs.

**Is the rationale for, and objectives of, the study clearly described?**

 The rationale for the review is clear and I agree with the thinking.

 The review has the following objective:

 “What are the most recent guideline update processes, including up-to-date prioritization methods, used by international or national groups who provide methods guidance for developing and updating clinical guidelines?”

 The authors indicate that the intent is a descriptive review, with findings presented as a narrative synthesis (page 6). However, the abstract states that the review findings “… will support clinical guideline developers to ensure appropriate investment of resources”. This could imply some kind of evaluation or recommendation coming from this review.

 I don’t think the latter is the intention of the authors, and I do think there is value in a purely descriptive review: if so, I would suggest slightly modifying for clarity the last sentence of the abstract to avoid misunderstanding. If, actually, there is a thought to going further with the results, for example to highlight evaluations that indicate update approaches that are somehow more ‘effective’ than others, then the review question needs to signal this too.

 I’m not familiar with the CIMO framework used to develop the review question, and I could not access the full source reference content. The framework seems quite logical in itself but I was confused by how the review details were mapped across to the domains in Table 1. I think that’s a fairly minor point, but I did note that the ‘Outcome’ domain includes “Evaluation of the process” and items that do actually seem to suggest some kind of judgement emerging from the review.

 I’m not sure that Table 1 adds anything to the protocol and it may provide confusion to readers unfamiliar with the framework.

**Is the study design appropriate for the research question?**

 The general review design is appropriate. I note a few points below.

Time frame

 (First, I diplomatically point out that the period 2011-2021 probably covers eleven years, not ten.)

 I agree with the reasoning for focusing on more recent guidance. Given the attention that this topic has been attracting in the last couple of years, and the emergence of new technologies such as AI in the review field, could there be a case for shifting the ten years forward, so starting with 2013 or 2014 and moving the end date even to 2023?

Eligibility criteria

 Reading the Methods as a whole, I am unclear about the scope of the review in terms of the kinds of organisations whose guidance would be eligible.

 Working from the definition on p3 - “… CGs are systematically developed statements … to assist practitioner and service users’ decisions about appropriate healthcare for specific clinical circumstances across the entire clinical system" – I take ‘clinical practice’ to include rehabilitation and preventive care, etc. This sets a wider scope for eligibility of organisations developing CGs than is indicated in Table 2. I was surprised to see national entities such as the UK Screening Committee and the Canadian Task Force for Preventive Health Care excluded both from this review and from that by Vernooij *et al* 2014 (being used as an index document).

 Also, the unit of analysis for the review is (I think) ‘update guidance developed by an organisation’. I did not fully understand how this translates over in practice to ‘disease-specific publications’ in a way that leads to exclusion. Does this mean that major organisations that focus on, e.g., cancer or cardiovascular disease would be excluded? If so, I think the review could miss relevant data.

Data extraction

 I reviewed Supplementary file 2. Overall, the data extraction form makes sense. This will very much be a text-based exercise, consistent with a narrative review approach. CG recommendations are generally based on finely-balanced judgements of benefits versus harms and costs. Updates may be driven by factors other than changing clinical effectiveness evidence. The form may need to be adaptable to accommodate the full spectrum of thinking presented by guideline organisations.

Data synthesis

 The details given are very brief and high level, but this may be appropriate at this stage. I comment above on the nature of the data that will be extracted. The analysis will be in keeping with a qualitative synthesis approach. I assume that the ‘evaluation of the process’ means a description of the evaluations that are identified, and their findings, rather than an evaluation by the authors of which processes appear to be most effective (see comments above).

**Are sufficient details of the methods provided to allow replication by others?**

 Yes, for the main part. The data synthesis methods are rather high level.

**Are the datasets clearly presented in a useable and accessible format?**

 Not applicable

**Overall**

 This is a generally well-written protocol for a review that is not in any way ‘cookie cutter’. This is a tricky review as the ‘data’ will not be standardised or lend themselves to a simple analysis. The topic is important and is likely to be of interest to a wide range of audiences.

Is the study design appropriate for the research question?

Yes

Is the rationale for, and objectives of, the study clearly described?

Yes

Are sufficient details of the methods provided to allow replication by others?

Yes

Are the datasets clearly presented in a useable and accessible format?

Not applicable

Reviewer Expertise:

Public health, health services research, clinical guideline development

I confirm that I have read this submission and believe that I have an appropriate level of expertise to confirm that it is of an acceptable scientific standard, however I have significant reservations, as outlined above.

# Response

**Published:** 2026-10-16

**Authors:** Karen Cardwell

**Affiliations:** Health Information and Quality Authority, Ireland

This manuscript describes the protocol for a systematic review of the methods used, by organisations developing clinical guidelines (CGs), for updating their recommendations. This review will be useful because CGs are dependent on an evidence base that may be changing in significant ways, with implications for health outcomes and resource use. Updates themselves require significant resources, so understanding how to prioritise them and carry them out efficiently and to good effect is important.

 The proposed review will focus on the most recent timeframe, and include guidance by major CG organisations internationally as well as peer reviewed reports. The analysis will be descriptive and narrative and offer useful insights to users, funders and those who develop CGs.

**Is the rationale for, and objectives of, the study clearly described?**

1. The rationale for the review is clear and I agree with the thinking. The review has the following objective: “What are the most recent guideline update processes, including up-to-date prioritization methods, used by international or national groups who provide methods guidance for developing and updating clinical guidelines?” The authors indicate that the intent is a descriptive review, with findings presented as a narrative synthesis (page 6). However, the abstract states that the review findings “… will support clinical guideline developers to ensure appropriate investment of resources”. This could imply some kind of evaluation or recommendation coming from this review. I don’t think the latter is the intention of the authors, and I do think there is value in a purely descriptive review: if so, I would suggest slightly modifying for clarity the last sentence of the abstract to avoid misunderstanding. If, actually, there is a thought to going further with the results, for example to highlight evaluations that indicate update approaches that are somehow more ‘effective’ than others, then the review question needs to signal this too.

We have updated this sentence in the abstract and introduction to say “This may support guideline development groups nationally and internationally in consideration of amendments to the current update processes.”

1. I’m not familiar with the CIMO framework used to develop the review question, and I could not access the full source reference content. The framework seems quite logical in itself but I was confused by how the review details were mapped across to the domains in Table 1. I think that’s a fairly minor point, but I did note that the ‘Outcome’ domain includes “Evaluation of the process” and items that do actually seem to suggest some kind of judgement emerging from the review. I’m not sure that Table 1 adds anything to the protocol and it may provide confusion to readers unfamiliar with the framework.

Thank you for this feedback. Please see response 4 to reviewer 1 above.

**Is the study design appropriate for the research question?**

 The general review design is appropriate. I note a few points below.

*Time frame*

1. First, I diplomatically point out that the period 2011-2021 probably covers eleven years, not ten.)

Thank you, we have updated this to eleven years.

1. I agree with the reasoning for focusing on more recent guidance. Given the attention that this topic has been attracting in the last couple of years, and the emergence of new technologies such as AI in the review field, could there be a case for shifting the ten years forward, so starting with 2013 or 2014 and moving the end date even to 2023?

We agree that the emergence of new technologies such as AI is an interesting field. Unfortunately, we are unable to shift the focus forward in time as the systematic review ^1^ has already been published but we appreciate this suggestion and will consider this going forward.

*Eligibility criteria*

1. Reading the Methods as a whole, I am unclear about the scope of the review in terms of the kinds of organisations whose guidance would be eligible. Working from the definition on p3 - “… CGs are systematically developed statements … to assist practitioner and service users’ decisions about appropriate healthcare for specific clinical circumstances across the entire clinical system" – I take ‘clinical practice’ to include rehabilitation and preventive care, etc. This sets a wider scope for eligibility of organisations developing CGs than is indicated in Table 2. I was surprised to see national entities such as the UK Screening Committee and the Canadian Task Force for Preventive Health Care excluded both from this review and from that by Vernooij et al 2014 (being used as an index document). Also, the unit of analysis for the review is (I think) ‘update guidance developed by an organisation’. I did not fully understand how this translates over in practice to ‘disease-specific publications’ in a way that leads to exclusion. Does this mean that major organisations that focus on, e.g., cancer or cardiovascular disease would be excluded? If so, I think the review could miss relevant data.

Yes, clinical guidelines relating to rehabilitation and preventative care were eligible for inclusion, we have specified this in the eligibility criteria in the updated manuscript. Disease-specific clinical guidelines were not eligible for inclusion and as such, major organisations that focus on specific disease such as cancer and cardiovascular diseases were not eligible for inclusion. This was noted as a limitation in the published systematic review. ^1^

*Data extraction*

1. I reviewed Supplementary file 2. Overall, the data extraction form makes sense. This will very much be a text-based exercise, consistent with a narrative review approach. CG recommendations are generally based on finely-balanced judgements of benefits versus harms and costs. Updates may be driven by factors other than changing clinical effectiveness evidence. The form may need to be adaptable to accommodate the full spectrum of thinking presented by guideline organisations.

Thank you for this recommendation, we will consider this in future clinical guideline related work undertaken by our research team.

*Data synthesis*

1. The details given are very brief and high level, but this may be appropriate at this stage. I comment above on the nature of the data that will be extracted. The analysis will be in keeping with a qualitative synthesis approach. I assume that the ‘evaluation of the process’ means a description of the evaluations that are identified, and their findings, rather than an evaluation by the authors of which processes appear to be most effective (see comments above).

Yes, this is correct. The evaluation of the process refers to any description of the evaluations identified, for example, the usability and or critique of the updating methodology, this example has been included in the updated manuscript.

**Are sufficient details of the methods provided to allow replication by others?**

 Yes, for the main part. The data synthesis methods are rather high level.

**Are the datasets clearly presented in a useable and accessible format?**

 Not applicable

**Overall**

 This is a generally well-written protocol for a review that is not in any way ‘cookie cutter’. This is a tricky review as the ‘data’ will not be standardised or lend themselves to a simple analysis. The topic is important and is likely to be of interest to a wide range of audiences.

**Is the rationale for, and objectives of, the study clearly described?**

 Yes

**Is the study design appropriate for the research question?**

 Yes

**Are sufficient details of the methods provided to allow replication by others?**

 Yes

**Are the datasets clearly presented in a useable and accessible format?**

 Not applicable

**Competing Interests**

 No competing interests were disclosed.

**Reviewer Expertise**

 Public health, health services research, clinical guideline development

 1. Cardwell, K., Quigley, J., Clyne, B., Tyner, B., Carrigan, M., Smith, S. M., Lynch, R., Hughes, C., Bradley, D., Kinsella, M., Holland, D., Ryan, M., & O’Neill, M. (2023). Systematic review finds processes used internationally, to update clinical guidelines, lack consistency and detail. Evidence & Policy, 19(4), 572-590. Retrieved Oct 16, 2026, from https://doi.org/10.1332/174426421X16854447463061

# Reviewer report: Reviewer response for version 1

**DOI:** 10.21956/hrbopenres.14656.r31006

**Published:** 2022-01-11

**Authors:** Julian Little

**Affiliations:** 1 School of Epidemiology and Public Health, University of Ottawa, Ottawa, ON, Canada

A systematic review of processes for updating guidelines will be a valuable piece of work. The protocol is well written and clear. Here are a number of comments for consideration:

1. Reference is made to “national clinical guidelines” in the abstract and the first paragraph of the introduction. However, the remit of the proposed work seems broader. Guidelines may be in place for jurisdictions that are sub-national, and for professional organizations. In addition, I was left uncertain whether preventive health care and public health were within scope.
2. In the second paragraph of the introduction, signal detection methods have been proposed for triggering updates of evidence syntheses that underpin guidelines. See for example Newberry SJ *et al.* ^1^, Shekelle PG *et al.* ^2^. Briefly showing the readers that these have been considered in developing the searches in the systematic review will increase confidence in the evidence as to whether or not these methods have been used; if they have been used, how much and what has been the experience; if there is no evidence that they have been used, is there any on why not.
3. I was surprised that there was no mention of the concept of living guidelines – see Akl EA *et al.* ^3^ (the same comment as for the signal detection methods; the COVID-19 pandemic has catalyzed interest) ****or the AGREE statement ^4^ ( https://www.agreetrust.org/agree-ii/), which includes a point about updating procedures (domain 3, point 14). The AGREE II instrument is a tool to assess the quality and reporting of practice guidelines and is internationally used. Having guidelines based on up-to-date evidence is obviously an important domain of guideline quality, and I think it's important to make the link to this in the review.
4. Table 1 – what belongs under “Mechanism” and under “Outcome” is unclear.
5. Table 2 – consideration might be given to Canadian and US Task Forces on Preventive Health Care, the UK National Screening Committee, and the IARC Cancer Prevention Handbooks (e.g. cervical cancer screening is covered in Vol. 10 and the upcoming Vol. 18; breast cancer in Vols. 7 and 15). This links back to the comments I made on the scope of the review. If preventive health is within scope, these strike me as well recognized international sources to consider.
6. There has been a large investment in clinical research in China, and there are many clinical guidelines ^5^. I think this work needs at least to be recognized. There are Chinese databases, e.g. CKNI and WanFang (see Cohen JF *et al.* ^6^). China accounts for more than 18% of the world’s population. There are also large proportions in India and in African countries, but there is less investment in clinical research. There has been a lot of activity in clinical guideline development in China ^5^. I do not have comprehensive knowledge of guidelines in China, but having seen a few, my sense is that they consider Chinese evidence, evidence from other jurisdictions, and also consider Chinese traditional medicine, so they have a mixture of unique attributes and those common to other jurisdictions. I’m not sure how much the Chinese guidelines will be picked up in the search strategy proposed in the protocol, which is why I suggested the Chinese databases. It may be that the investigators decide that investigating Chinese guidelines other than what would be picked up in the search strategy presented in the protocol is out of scope, but I think it's important to be clear in reporting this decision and the rationale for it. The guideline development in India ^7^ and Africa ^8^ (this reference on hypertension guidelines may be helpful) may in large part be based on adapting guidelines from other jurisdictions, and thus be more likely to be picked up in the strategy in the protocol.

Is the study design appropriate for the research question?

Yes

Is the rationale for, and objectives of, the study clearly described?

Yes

Are sufficient details of the methods provided to allow replication by others?

Yes

Are the datasets clearly presented in a useable and accessible format?

Not applicable

Reviewer Expertise:

Epidemiology and Prevention of Congenital Anomalies; Psychosis and affective psychosis; Cancer Epidemiology and Prevention; Molecular and Human Genome Epidemiology; Evidence synthesis related to public health; Health services research

I confirm that I have read this submission and believe that I have an appropriate level of expertise to confirm that it is of an acceptable scientific standard, however I have significant reservations, as outlined above.

## References

[1] : Surveillance and Identification of Signals for Updating Systematic Reviews: Implementation and Early Experience. 23865098

[2] : Assessment of a method to detect signals for updating systematic reviews. *Syst Rev* .2014;3: 10.1186/2046-4053-3-1313 24529068 10.1186/2046-4053-3-13 PMC3937021

[3] : Living systematic reviews: 4. Living guideline recommendations. *J Clin Epidemiol* .2017;91: 10.1016/j.jclinepi.2017.08.00947-53 28911999 10.1016/j.jclinepi.2017.08.009

[4] : The AGREE Reporting Checklist: a tool to improve reporting of clinical practice guidelines. *BMJ* .2016;352: 10.1136/bmj.i1152i1152 26957104 10.1136/bmj.i1152 PMC5118873

[5] : Clinical practice guidelines in China. *BMJ* .2018; 10.1136/bmj.j5158 10.1136/bmj.j5158 PMC579798229437564

[6] : Should we search Chinese biomedical databases when performing systematic reviews?. *Syst Rev* .2015;4: 10.1186/s13643-015-0017-323 25874584 10.1186/s13643-015-0017-3 PMC4374381

[7] : Adapting clinical guidelines in India—a pragmatic approach. *BMJ* .2017; 10.1136/bmj.j5147 10.1136/bmj.j5147 PMC569155429150419

[8] : Evaluation of all African clinical practice guidelines for hypertension: Quality and opportunities for improvement. *J Eval Clin Pract* .2019;25(4) : 10.1111/jep.12954565-574 29901241 10.1111/jep.12954

# Response

**Published:** 2026-10-16

**Authors:** Karen Cardwell

**Affiliations:** Health Information and Quality Authority, Ireland

A systematic review of processes for updating guidelines will be a valuable piece of work. The protocol is well written and clear. Here are a number of comments for consideration:

1. Reference is made to “national clinical guidelines” in the abstract and the first paragraph of the introduction. However, the remit of the proposed work seems broader. Guidelines may be in place for jurisdictions that are sub-national, and for professional organizations. In addition, I was left uncertain whether preventive health care and public health were within scope.

We have removed the word “National” in the abstract. We do refer to “National Clinical Guidelines” in the first sentence of the introduction, but this is to set the context for this piece of work. With respect to the inclusion of preventive and public health care publications, we have specified in the inclusion criteria that these are eligible for inclusion.

1. In the second paragraph of the introduction, signal detection methods have been proposed for triggering updates of evidence syntheses that underpin guidelines. See for example Newberry SJ et al. 1, Shekelle PG et al. 2. Briefly showing the readers that these have been considered in developing the searches in the systematic review will increase confidence in the evidence as to whether or not these methods have been used; if they have been used, how much and what has been the experience; if there is no evidence that they have been used, is there any on why not.

Thank you for this suggestion. In the second paragraph of the introduction we acknowledge that the currency of the evidence underpinning a clinical guideline is one aspect that may indicate the need to update a clinical guideline. We have subsequently added an appropriate reference to reflect this.

1. I was surprised that there was no mention of the concept of living guidelines – see Akl EA et al. 3 (the same comment as for the signal detection methods; the COVID-19 pandemic has catalyzed interest) or the AGREE statement4 (https://www.agreetrust.org/agree-ii/), which includes a point about updating procedures (domain 3, point 14). The AGREE II instrument is a tool to assess the quality and reporting of practice guidelines and is internationally used. Having guidelines based on up-to-date evidence is obviously an important domain of guideline quality, and I think it's important to make the link to this in the review.

Living guidelines were considered during the conduct of this review. They are cited in the introduction and following piloting, the data extraction form was refined to include a specific section on living guidelines, and relevant data were extracted where reported. This detail is described in the published systematic review ^1^ arising from this protocol. The focus of this review was on clinical guideline update processes rather than guideline quality assessment. However, we acknowledge the relevance of AGREE II and its emphasis on updating procedures as an important component of guideline quality and have subsequently detailed this in the introduction along with an appropriate citation.

1. Table 1 – what belongs under “Mechanism” and under “Outcome” is unclear.

Following peer review feedback on the published systematic review ^1^ arising from this protocol, we now refer to a modified version of the CIMO framework and have removed the “mechanism” domain. The aim of this review was to describe the current guideline update processes used internationally, rather than exploring a realist synthesis type question of “what works, for whom, under what circumstances?” The CIMO framework was initially selected as our research question was not adequately addressed by a typical PICO framework. CIMO is often used in implementation science and the guideline process falls somewhere between implementation and clinical. The authors did not use the CIMO framework for synthesis, instead, a narrative synthesis was conducted. The CIMO framework was used to define the eligibility criteria of articles for inclusion in this review. As such, the CIMO framework was only referred to in the Screening and Data extraction sections of the manuscript. The authors appreciate that had this have been a realist review, the mechanisms could not have been pre-specified as an eligibility criterion, only hypothesised as part of an initial programme theory or theories. In our review we pre-specified the mechanism as “Clinical guidelines considered for updating (includes full, partial (also referred to as modular), rapid updates). Tools or guidance available to support prioritisation” to help when screening the literature. The authors are in agreement that mechanism on our paper does not conform to the ideas of ‘realist’ or ‘generative’ mechanism and so the authors have removed reference to mechanism in the eligibility criteria. The mechanism is now more appropriately captured with a more detailed description of the context and outcome. To avoid any confusion for the reader, the authors now refer to a modified version of the CIMO framework.

1. Table 2 – consideration might be given to Canadian and US Task Forces on Preventive Health Care, the UK National Screening Committee, and the IARC Cancer Prevention Handbooks (e.g. cervical cancer screening is covered in Vol. 10 and the upcoming Vol. 18; breast cancer in Vols. 7 and 15). This links back to the comments I made on the scope of the review. If preventive health is within scope, these strike me as well recognized international sources to consider.

Thank you for this feedback. As the systematic review ^1^ has already been completed and published, it is not possible to incorporate additional organisations. We agree these are important sources and we have noted these organisations as potential sources of information for future research.

1. There has been a large investment in clinical research in China, and there are many clinical guidelines5. I think this work needs at least to be recognized. There are Chinese databases, e.g. CKNI and WanFang (see Cohen JF et al.6). China accounts for more than 18% of the world’s population. There are also large proportions in India and in African countries, but there is less investment in clinical research. There has been a lot of activity in clinical guideline development in China5. I do not have comprehensive knowledge of guidelines in China, but having seen a few, my sense is that they consider Chinese evidence, evidence from other jurisdictions, and also consider Chinese traditional medicine, so they have a mixture of unique attributes and those common to other jurisdictions. I’m not sure how much the Chinese guidelines will be picked up in the search strategy proposed in the protocol, which is why I suggested the Chinese databases. It may be that the investigators decide that investigating Chinese guidelines other than what would be picked up in the search strategy presented in the protocol is out of scope, but I think it's important to be clear in reporting this decision and the rationale for it. The guideline development in India7 and Africa8 (this reference on hypertension guidelines may be helpful) may in large part be based on adapting guidelines from other jurisdictions, and thus be more likely to be picked up in the strategy in the protocol.

Thank you for this feedback. As mentioned above, the systematic review ^1^ has already been published and in the context of when this work was reported, we also did not have comprehensive knowledge of guideline development in China. As such, we do not feel it appropriate to reference it in the protocol but we have noted this as an important consideration going forward.

**Is the rationale for, and objectives of, the study clearly described?**

 Yes

**Is the study design appropriate for the research question?**

 Yes

**Are sufficient details of the methods provided to allow replication by others?**

 Yes

**Are the datasets clearly presented in a useable and accessible format?**

 Not applicable

**References**

 1. Newberry SJ, Ahmadzai N, Motala A, Tsertsvadze A, et al.: Surveillance and Identification of Signals for Updating Systematic Reviews: Implementation and Early Experience. PubMed Abstract

 2. Shekelle PG, Motala A, Johnsen B, Newberry SJ: Assessment of a method to detect signals for updating systematic reviews.Syst Rev. 2014; 3: 13 PubMed Abstract | Publisher Full Text

 3. Akl EA, Meerpohl JJ, Elliott J, Kahale LA, et al.: Living systematic reviews: 4. Living guideline recommendations.J Clin Epidemiol. 2017; 91: 47-53 PubMed Abstract | Publisher Full Text

 4. Brouwers MC, Kerkvliet K, Spithoff K, AGREE Next Steps Consortium: The AGREE Reporting Checklist: a tool to improve reporting of clinical practice guidelines.BMJ. 2016; 352: i1152 PubMed Abstract | Publisher Full Text

 5. Chen Y, Wang C, Shang H, Yang K, et al.: Clinical practice guidelines in China. BMJ. 2018. Publisher Full Text

 6. Cohen JF, Korevaar DA, Wang J, Spijker R, et al.: Should we search Chinese biomedical databases when performing systematic reviews?. Syst Rev. 2015; 4: 23 PubMed Abstract | Publisher Full Text

 7. Mehndiratta A, Sharma S, Gupta N, Sankar M, et al.: Adapting clinical guidelines in India—a pragmatic approach. BMJ. 2017. Publisher Full Text

 8. Okwen PM, Maweu I, Grimmer K, Margarita Dizon J: Evaluation of all African clinical practice guidelines for hypertension: Quality and opportunities for improvement.J Eval Clin Pract. 2019; 25 (4): 565-574 PubMed Abstract | Publisher Full Text

**Competing Interests**

 No competing interests were disclosed.

**Reviewer Expertise**

 Epidemiology and Prevention of Congenital Anomalies; Psychosis and affective psychosis; Cancer Epidemiology and Prevention; Molecular and Human Genome Epidemiology; Evidence synthesis related to public health; Health services research

 1. Cardwell, K., Quigley, J., Clyne, B., Tyner, B., Carrigan, M., Smith, S. M., Lynch, R., Hughes, C., Bradley, D., Kinsella, M., Holland, D., Ryan, M., & O’Neill, M. (2023). Systematic review finds processes used internationally, to update clinical guidelines, lack consistency and detail. Evidence & Policy, 19(4), 572-590. Retrieved Oct 16, 2026, from https://doi.org/10.1332/174426421X16854447463061
